# Preparation, mechanics and self-sensing performance of sprayed reactive powder concrete

**DOI:** 10.1038/s41598-022-11836-y

**Published:** 2022-05-12

**Authors:** Yunlong Zhang, Jianxin Wang, Jing Wang, Xuesong Qian

**Affiliations:** 1 School of Transportation Science and Engineering, Jilin Jianzhu University, Xincheng Street, Changchun, 130118 Jilin China; 2 Research Center of Traffic Disaster Prevention and Mitigation Jilin Jianzhu University, Jilin Jianzhu University, Xincheng Street, Changchun, 130118 Jilin China; 3 Key Laboratory for Comprehensive Energy Saving of Cold Regions Architecture of Ministry of Education, Jilin Jianzhu University, Xincheng Street, Changchun, 130118 Jilin China

**Keywords:** Engineering, Civil engineering, Materials science, Structural materials, Ceramics, Composites, Mechanical properties

## Abstract

The emergence of shotcrete provides a new idea for construction methods, but with the development of society, the traditional shotcrete has been unable to meet the needs of structure. Therefore, concrete with better material properties is needed to replace traditional shotcrete. Reactive powder concrete (RPC) is a well-known ultrahigh strength concrete and widely used. Its material properties are better than shotcrete. However, the sprayable performance of RPC and the properties of this sprayed materials have not been reported. Therefore, to make up for the deficiency of ordinary shotcrete, the material properties of sprayed RPC were studied in depth. Response surface method was used to study the effects of different silica fume content, fly ash content and steel fiber volume content on workability, mechanical properties and crack sensitivity. The sprayed reactive powder concrete (sprayed RPC) was proposed for the first time. All models were reliable through variance analysis. The performance of sprayed RPC was better when the workability was between 140 and 160 mm. When the silica fume/binder ratio was 15%, the fly ash/binder ratio was 13.203%, and the volume content of steel fibers was 2%, the mechanical properties and crack sensitivity of sprayed RPC can reach a satisfactory degree. By studying the workability, mechanical properties and crack sensitivity of sprayed RPC, the optimum mix ratio of sprayed RPC was obtained. Steel fiber sprayed RPC can detect structural damage. Results lay the foundation for popularization and application to practical engineering.

## Introduction

Shotcrete is a special technology for placing concrete. Although the construction is flexible, convenient and widely used^1,2^, its strength is low. To improve the performance of shotcrete, scholars studied the high-performance shotcrete through different methods^3–5^. But high-quality coarse aggregate is lacking in many parts of the world^6^, so, the production of high-performance shotcrete has always been a challenging task in the application of shotcrete. Furthermore, the structural integrity may decrease with service life. Therefore, a kind of high-performance shotcrete with self-monitoring performance is necessary.

Reactive powder concrete (RPC) is a kind of material with excellent mechanical properties and durability. Coarse aggregate is eliminated in material selection, and optimization is conducted at nanometer and micron levels^6^. However, no reports have been found regarding the application of RPC in shotcrete. Therefore, combining RPC and injection technology to explore a sprayable RPC is of technical innovation value. At present, researchers improve RPC performance through fiber reinforcement and mix ratio optimization. In terms of fiber reinforcement, the fibers most often studied by scholars are steel fibers, because it has relatively high tensile strength, elastic modulus, and corrosion resistance^7^. Some research reports indicate that steel fiber content has a great influence on RPC performance^8,9^. When the content of steel fibers is 0–6%, the 28d compressive strength of RPC can be increased by 15–40%^10–12^ and the splitting strength can be increased by 60%–300%^11,13^. The incorporation of too much steel fibers is not conducive to the workability of RPC^10,12,14^. Workability is a key index for evaluating the spray performance^15^, so, it is necessary to further study the steel fiber content in sprayed RPC.

In terms of optimizing the matrix mix, silica fume can effectively improve the mechanical properties of RPC^6,16–18^. Zemei Wu et al.^17^ studied that when the content of silica fume within 0%–25%, the maximum compressive strength of RPC can reach 120 MPa and the maximum tensile strength can be increased by 85% . Other studies have shown that fly ash can also improve the performance of RPC^9,19,20^. Tiefeng Chen et al.^9^ studied the influence of fly ash with different contents (0%–30%) on the compressive strength of UHPC and pointed out that the incorporation of 20% fly ash could increase the compressive strength of UHPC by a maximum of 10%. Fly ash can improve the fluidity of the cement paste^21^. In addition, the mixed incorporation of silica fume and fly ash into cement-based materials has better mechanical properties than the single incorporation^22,23^. Therefore, considering the workability and mechanical properties of sprayed RPC, it is necessary to study its mixing ratio.

To ensure the safety of using sprayed RPC, it is very important to accurately monitor the structural integrity of the structure. However, traditional sensors have limited detection range and may lead to structural defects. To overcome the shortcomings of traditional sensors, cement-based sensors have been widely studied. Cement-based sensors are made of cementitious materials and have self-sensing properties, this ability allows it to respond to changes in itself^24^. When cracks occur in the structure, the resistivity will increase due to the extension of the propagation path of the radio wave^25^, so we can monitor the safety of the structure according to the change of the resistivity. Cement-based sensors used in the structure can not only monitor a wide range, but also ensure that the strength of the structure is not destroyed. Adding conductive materials into concrete can reduce resistivity and achieve higher precision self-sensing^26^. Some conductive materials can not only enhance the self-sensing ability of cement-based sensors, but also enhance the strength and durability of the material, such as steel fibers^27,28^. Steel fiber reinforced cement-based sensors have been reported to be thousands of times more sensitive than commercial strain gauges^29^. At present, some researchers have studied the electrical properties of steel fiber cement-based materials^30–32^. The cement-based materials with steel fiber content of 1% show high self-sensing ability and can be used to detect tensile strain (or stress) and damage (or crack) of structural members^31^. However, the research on damage sensing capability of sprayed RPC has not been found. Therefore, to monitor the structural integrity in the actual application of sprayed RPC, it is of great significance to study the damage perception ability of sprayed RPC.

In conclusion, to make up for the defect of strength shortage of ordinary shotcrete material, RPC is applied to the field of shotcrete and its material performance need be studied. To monitor the safety of the structure in the application process of sprayed RPC, it is very important to study the self-sensing properties of sprayed RPC. However, there are no relevant reports on the material performance of sprayed RPC. Therefore, this study aimed to develop a sprayed RPC that can guarantee some mechanical properties and can monitor damage (crack). This study investigated the effects of matrix mix factor and steel fiber volume content on sprayed RPC. The relationship between three process variables (silica fume to binder ratio, fly ash to binder ratio, and steel fiber volume content) along with mechanical properties and crack sensitivity of sprayed RPC were modeled using the response surface method (RSM). On the basis of the model, the influence degree and rule of process variables on compressive strength, splitting tensile strength and crack sensitivity were determined, and the optimal amount was obtained. The results provide basis for the practical application of sprayed RPC.

## Materials and methods

### Raw material

The P.II 52.5 cement, Silica fume and fly ash were used in this study. Their quality inspection reports were provided by the manufacturer and complied with the General Portland Cement Testing Standard (GB 175–2007)^33^ and the technical specifications of the application of mineral admixtures(GB/T 51,003/2014)^34^. The chemical and physical properties of these materials are summarized in Table 1. The superplasticizer used was a star-shaped polycarboxylic acid high-performance superplasticizer and the water-reducing rate of it exceeded 28%. Manufactured sand was used as fine aggregate, its fineness modulus is 3.6. Considering the peak bearing capacity^13^ and the nozzle diameter, copper-plated steel fibers with diameter of 0.2 mm, length of 8 mm and tensile strength of 2907 MPa were selected.

**Table 1:** The properties of cement, silica fume and fly ash.

Properties	Cement		Silica fume		Fly ash	
	Standard value	Actual value	Standard value	Actual value	Standard value	Actual value
Loss on ignition (%)	≤ 3.5	1.72	≤ 2	1.5	≤ 8	0.77
MgO (%)	≤ 5	0.99	–	–	–	–
SO_3_ (%)	≤ 3.5	2.56	–	–	≤ 3	0.1
Insolubles (%)	≤ 1.5	0.9	–	–	–	–
Cl^-^(%)	≤ 0.06	0.008	≤ 2	0.8	–	–
SiO_2_(%)	–	–	≥ 94	94.24	–	–
PH	–	–	4–8.5	7	–	–
Moisture content (%)	–	–	≤ 3	0.9	≤ 1	0.11
Water demand ratio (%)	–	–	≤ 125	119	≤ 105	99
CaO_3_	–	–	–	–	≤ 1	0.68
Fineness (%)	–	–	–	–	≤ 30	14.2
Stability (Reye method) (mm)	–	–	–	–	–	3
Intensity activity index (%)	–	–	–	–	≤ 70	74

The water to binder ratio was fixed at 0.2, and the sand to binder ratio was fixed at 0.7. The mixing amount of superplasticizer was adjusted according to the spray property of the mixture. Finally, the content of superplasticizer was determined to be 1% of the mass of the cementitious material. The specific mix of the test is shown in Table 2.

**Table 2:** The mix proportions of sprayed reactive powder concrete (kg/m^3^).

Mix	Cement	Silica fume	Fly ash	Manufactured sand	Water	Steel fiber	Superplasticizer
B-1	928.40	185.68	123.79	866.51	247.57	117	13.62
B-2	791.68	304.49	121.80	852.58	243.60	117	13.40
B-3	798.49	184.27	245.69	859.91	245.69	117	13.51
B-4	664.86	302.21	241.77	846.19	241.77	117	13.30
B-5	863.20	184.97	184.97	863.20	246.63	78	13.56
B-6	728.03	303.35	182.01	849.37	242.68	78	13.35
B-7	863.20	184.97	184.97	863.20	246.63	156	13.56
B-8	728.03	303.35	182.01	849.37	242.68	156	13.35
B-9	859.49	245.57	122.78	859.49	245.57	78	13.51
B-10	731.14	243.71	243.71	852.99	243.71	78	13.40
B-11	859.49	245.57	122.78	859.49	245.57	156	13.51
B-12	731.14	243.71	243.71	852.99	243.71	156	13.40
B-13	795.07	244.64	183.48	856.23	244.64	117	13.46
B-14	795.07	244.64	183.48	856.23	244.64	117	13.46
B-15	795.07	244.64	183.48	856.23	244.64	117	13.46
B-16	795.07	244.64	183.48	856.23	244.64	117	13.46
B-17	795.07	244.64	183.48	856.23	244.64	117	13.46

### Fabrication of sprayed RPC

To eliminate the interference of superplasticizer on material properties, the amount of superplasticizer was fixed. Because the superplasticizer according to the stirring time to gradually play the effect, the workability of sprayed RPC was controlled by stirring time. The wet mixed sprayed RPC can ensure both ejection and adhesion.

The specific preparation process of sprayed RPC was as follows. (1) The cement, silica fume, fly ash, and fine aggregate were mixed and dry mixed for120s. (2) Steel fiber is added, and the mixture was dry mixed for 120 s. (3) Water and superplasticizer were added to the mixture and stirred for 90–240 s. (4) The mixture was poured into the jet to prepare for injection.

**Figure 1:**
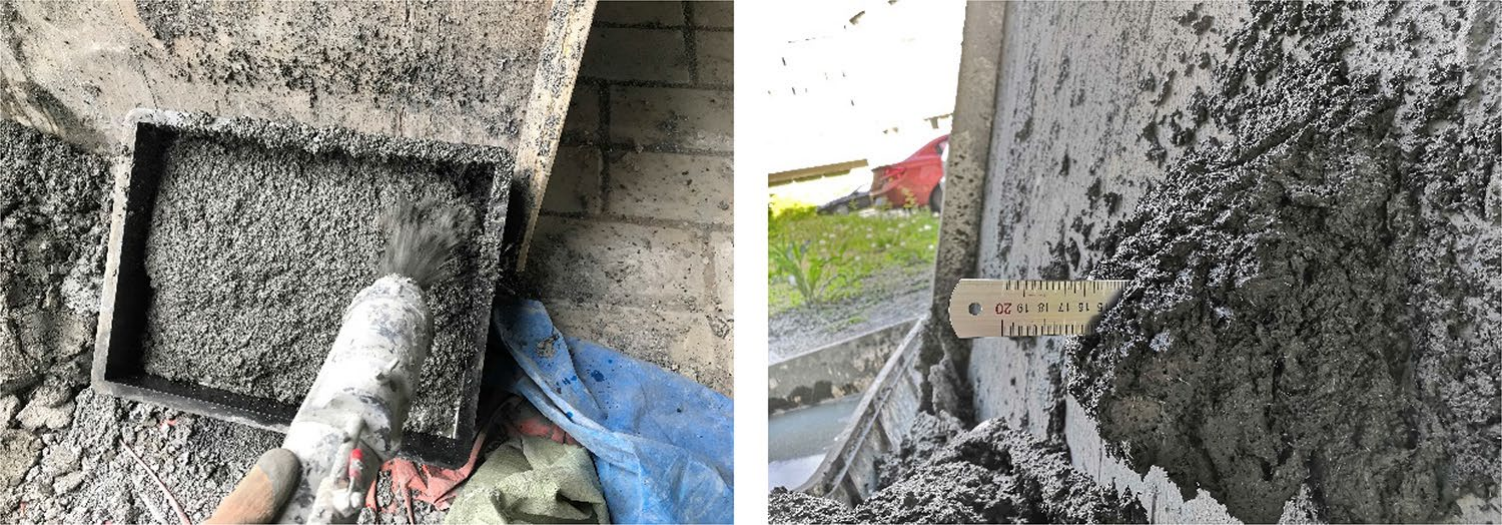
The tester performs the spray and thickness of single point injection.

The spray process is shown in Fig. 1. The inner diameter of the conveying pipe of the jet was 32 mm, the wind pressure was fixed at 8 MPa, the nozzle was 90° with the large plate mold and the spraying distance was less than 1 m. The included angle between the large plate and the ground was 45°.Under the condition that the plate was perpendicular to the ground, the single injection effect of the test point is shown in Fig. 1, and the thickness of single injection can reach 15 cm. It can prove that the sprayed RPC prepared by combining RPC mix ratio and spraying process has good spray-ability and bonding property.

The cube specimens of sprayed RPC were made by spraying by large plate method (350 mm × 450 mm × 120 mm). After spraying, all specimens were wrapped with plastic cloth and demolded after curing for 24 h. To simulate the curing conditions of tunnel, the strength test specimens were moved to the outdoor curing for 28d. The APS bituminous mixture automatic cutting machine and rock cutting machine were used to cut the large plate specimen into cubes of 100 mm × 100 mm dimensions for compressive and splitting tensile strength tests. The 100 mm × 100 mm × 400 mm prisms were cut for crack sensitivity tests.

### Experimental method

#### Box-Behnken design (BBD)

Response surface methodology (RSM) is a mathematical and statistical technique that employs both a graphical representation and numerical methods for developing response models between one or more independent variables and responses. Using the developed response models from RSM, multi-objective optimization has been successfully performed based on predefined goals for variables and responses^35^. And some published works on concrete technology have used RSM for model development and multi-objective optimization^36,37^.

Response surface methodology includes many design methods, among which, the Box-Behnken method is a response surface design that can evaluate the nonlinear relationship between indicators and factors. It does not require repeated testing. Under the same experimental factors, it is more economical and efficient, because it requires fewer experiments^21^.

In this paper, Box-Behnken design was established by using Design-expert12.0® software. The silica fume/binder ratio (*A*), fly ash/binder ratio (*B*), and volume content of steel fiber (*C*) were taken as process variables. The compressive strength(R1), splittiing tensile strength(R2) and crack sensitivity(R3) of the mixture were set as the response variables. The response surface model of 17 experimental points, namely, 12 factors and 5 center points, was established.

Table 3 shows the levels of experimental factors, and the corresponding code values. The low and high levels of each factor were respectively encoded as − 1 and + 1, whereas the central points were encoded as 0.

**Table 3:** Levels of experimental factors, given in actual and code values.

Symbol	Factors	Actual Values			Code Values		
A	Silica fume/binder ratio (%)	15	20	25	− 1	0	1
B	Fly ash/binder ratio (%)	10	15	20	− 1	0	1
C	Volume content of steel fibers (%)	1	1.5	2	− 1	0	1

#### Fluidity

**Figure 2:**
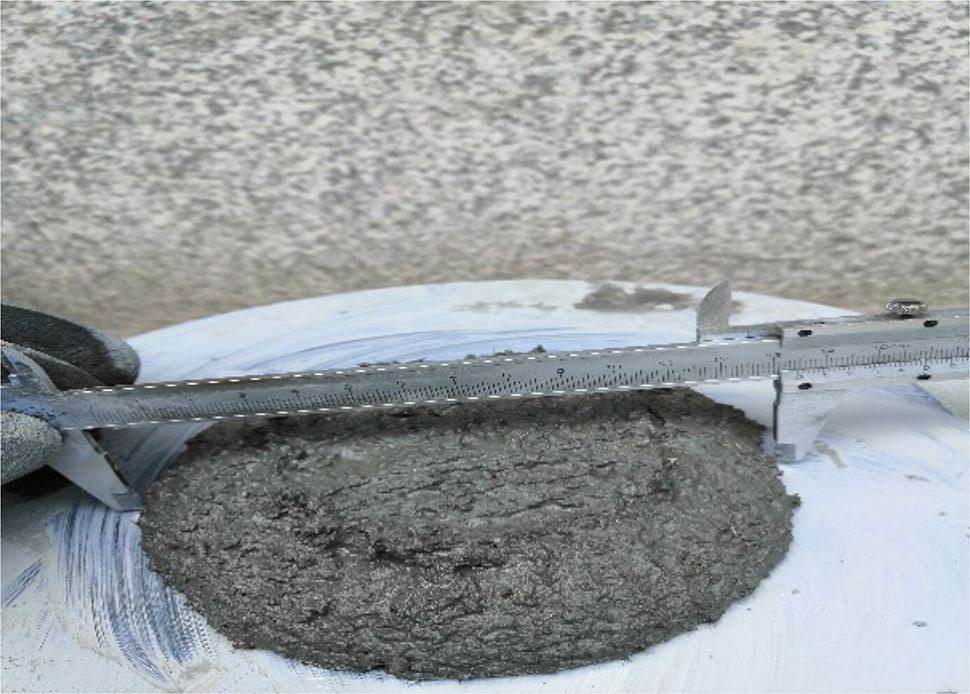
Jumping table test.

The jumping table method was used to measure the fluidity of the mixture. The mixture was divided into two layers and loaded into the flow test mold quickly. The first layer was tamped for 15 times, and the second layer was tamped for 10 times. After tamping, the mixture above the conical mold was scraped and wiped. The vibration started immediately, and 25 tampings were completed within 25 ± 1 s. After tamping, the maximum diffusion diameter and the diameter in the vertical direction of the bottom surface of the mixture were measured, and the average value was calculated to be accurate to 1 mm. This was the fluidity of the mixture. The whole process is completed within 6 min. Jumping table test is shown in Fig. 2.

Since no relevant reports have been found to study the fluidity of sprayed RPC, the relationship between fluidity and sprayed effect needs to be continuously tested. Finally, it is concluded that the sprayed effect of sprayed RPC with fluidity between 140 mm and 160 mm measured by jumping test is better, and the mixture has better bonding effect with the large plate.

#### Mechanical test

According to GB/T 31,387–2015 standard^38^, the specimen dimensions were selected as 100 mm × 100 mm × 100 mm to test the compressive strength. According to GB/T 50,080–2016^39^, specimen dimensions of 100 mm × 100 mm × 100 mm were selected to testing the splitting tensile strength. The YAR-2000 hydraulic press by New Test Machine, Ltd. (Changchun, China) was used to test the strength of specimen. The mean value of the three measurements were used as the compressive and splitting tensile strengths of the specimens. The loading rate of compression test was kept between 1.2 and 1.4 MPa s^−1^,and the loading rate of splitting tensile strength test was kept between 0.08 and 0.1 MPa s^−1^.

#### Crack sensitivity test

**Figure 3:**
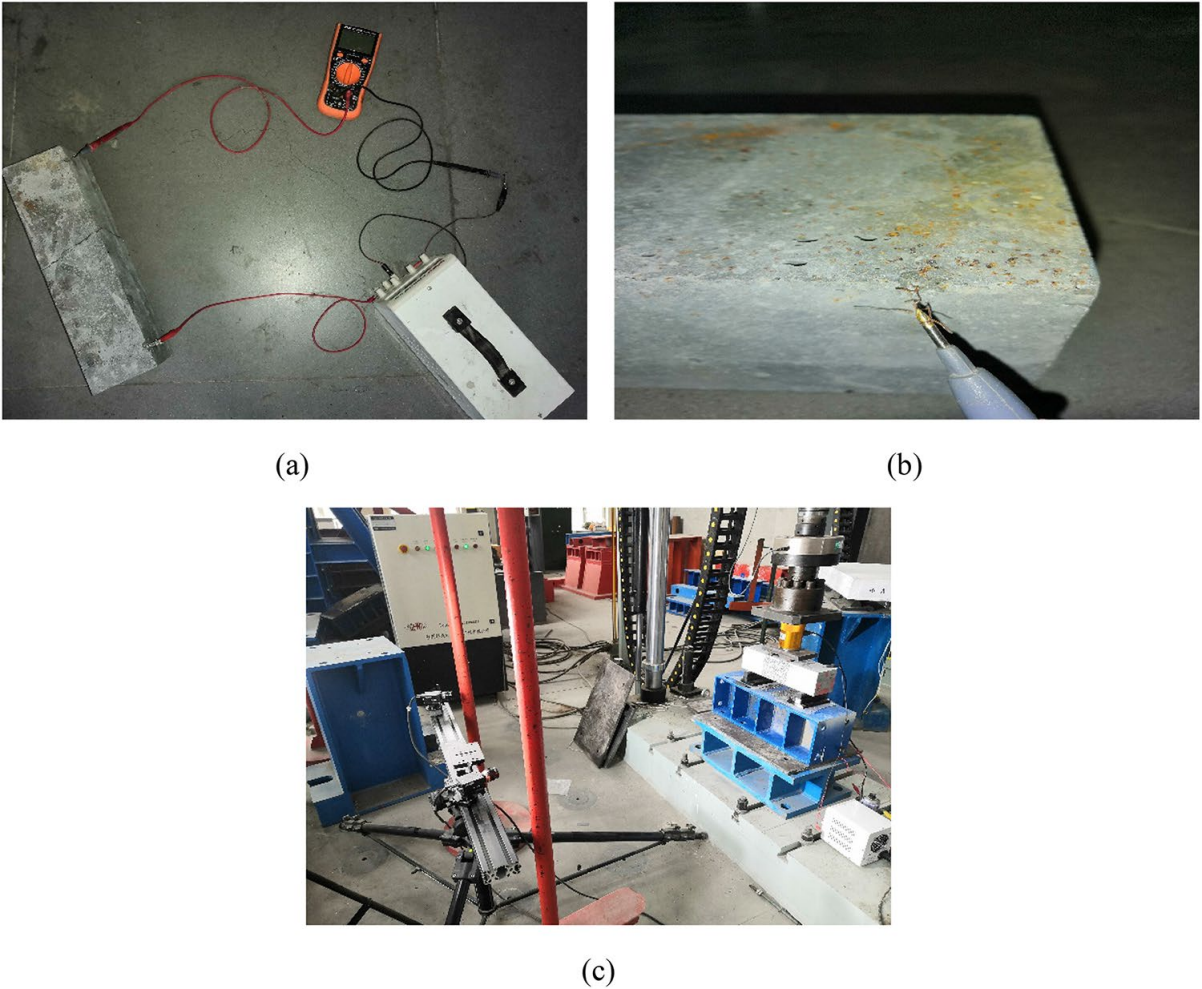
Test and instrument pictures.

Crack sensitivity is the percentage change in resistance per unit crack width (% mm^−1^). During the spray, two stainless steel gauze electrodes were embedded in the corresponding positions of large plate modes. The exposed electrodes were cut off, because all the surfaces of the sprayed RPC were required to be cut surfaces. The prism specimens with electrode dimensions of 100 mm × 100 mm × 400 mm were selected in the crack sensitivity test. The specimens were pretreated before the test, and small defects were made around the two electrodes, so that the electrodes could be exposed and connected to the power supply and the multimeter. Direct current (DC) stabilized power supply was selected as the test power source, and the voltage(*V*) was fixed as 5 V. The specific connection method is shown in Fig. 3a and b. The test process is shown in Fig. 3c. During the test, the current *I* passing through the specimen was measured at any time, and the resistance *R* of the specimen is calculated by Ohm’s law. Digital image correlation (DIC) technique was used to monitor the development of the crack width during bending. The specific calculation equation is as follows (Eq. (1)):

1 $$\begin{array}{*{20}c} {R = \frac{V}{I}} \\ \end{array}$$

The percentage of resistance change (R%) of the specimen is determined by Eq. (2), where *R*_*0*_ is the initial resistance of the specimen.

2 $$\begin{array}{*{20}c} {R\% = \left( {\frac{R}{{R_{0} }} - 1} \right) \times 100} \\ \end{array}$$

## Results and discussion

### Establishment and verification of response surface model

Table 4 reflects the mixing ratio of 17 experimental groups with sprayed RPC and the results of compressive strength, splitting tensile strength, and crack sensitivity after curing for 28d. Among them, B13–B17 were the five central experimental groups that were used to evaluate the stability of the model.

**Table 4:** Experimental factors and response results of the BBD matrix design.

Mix number	Code level of variables			Response values		
	A	B	C	R1: 28d Compressive strength (MPa)	R2: 28d Splitting tensile strength (MPa)	R3: Crack sensitivity (% mm^−1^)
B-1	− 1	− 1	0	66.40	9.38	12.75
B-2	1	− 1	0	70.00	7.75	12.05
B-3	− 1	1	0	98.61	8.24	14.06
B-4	1	1	0	85.79	8.21	9.87
B-5	− 1	0	− 1	97.73	8.08	36.12
B-6	1	0	− 1	113.80	8.73	46.27
B-7	− 1	0	1	114.79	11.23	9.81
B-8	1	0	1	99.58	8.22	7.91
B-9	0	− 1	− 1	115.86	9.62	15.61
B-10	0	1	− 1	100.49	8.50	11.94
B-11	0	− 1	1	119.28	8.44	11.89
B-12	0	1	1	113.27	10.41	1.68
B-13	0	0	0	118.02	10.28	11.30
B-14	0	0	0	105.50	9.32	11.13
B-15	0	0	0	109.66	9.86	15.51
B-16	0	0	0	94.13	9.25	9.86
B-17	0	0	0	111.24	8.33	11.81 ara>

The response surface model was established using multiple regression analysis method in Design Expert 12.0® software. Analysis of variance (ANOVA)was used to test the accuracy of the regression model and the significance of the influencing factors. Statistical parameters were used to verify the accuracy of the model, the specific parameters are shown in Table 5.

**Table 5:** Parameter estimation and variance analysis of the response surface model for bending performance and crack sensitivity.

Response	R1: 28d Compressive strength (MPa)			R2: 28d Split tensile strength (MPa)			R3: Crack sensitivity (% mm ^−1^)		
Transform	Inverse Sqrt			Natural Log			Power (Lambda = -0.2)		
R^2^	0.75			0.80			0.88		
	Coefficient estimate	*F*-value	Coefficient estimate	*F*-value	Coefficient estimate	*F*-value	Coefficient estimate	F-value	*p*-value
Intercept	0.0966	–	0.0966	–	0.0966	-	0.6145	–	–
A	1.55E-06	0.0416	1.55E-06	0.0416	1.55E-06	0.0416	0.0068	0.2413	0.6338
B	0.0001	1.98	0.0001	1.98	0.0001	1.98	0.0421	9.26	0.0124
C	9.01E-06	0.2413	9.01E-06	0.2413	9.01E-06	0.2413	0.0833	36.33	0.0001
AB	NS	NS	NS	NS	NS	NS	NS	NS	NS
AC	0.0001	1.37	0.0001	1.37	0.0001	1.37	NS	NS	NS
BC	NS	NS	NS	NS	NS	NS	0.065	11.05	0.0077
A^2^	0.0004	9.9	0.0004	9.9	0.0004	9.9	− 0.0573	9.08	0.013
B^2^	0.0002	5.22	0.0002	5.22	0.0002	5.22	0.0551	8.39	0.0159
C^2^	0.0003	8.76	0.0003	8.76 ara>	0.0003	8.76	NS	NS	NS
Model	–	3.81	–	3.81	–	3.81	–	12.24	0.0004
Lack of fit	–	3.18	–	3.18	–	3.18	–	5.57	0.0591

Where factor A is the silica fume/binder ratio, B is the fly ash/ binder ratio, C is the volume content of steel fiber. NS means that insignificant factors are ignored.

In the process of modeling, non-significant variables were judged and removed from the model according to the values of p and *R*^2^. Although some models were not significant (*p* > 0.05), they cannot be excluded considering the hierarchy of the models and the value of the model coefficient of determination *R*^2^. Considering that the *R*^2^ value of these responses were all greater than 0.75, a good correlation between model predictions and measured data was indicated.

The following equations (Eq. (3)–(5)) provides the mathematical models for compressive strength (R1, MPa), split tensile strength (R2, MPa) and crack sensitivity (R3, % mm − 1):

3 $$\begin{gathered} \frac{1}{{\sqrt {R1} }} = 0.281771 - 0.017046 \cdot A - 0.008773 \cdot B - 0.075085 \cdot C + 0.001429 \cdot AC \hfill \\ \quad \quad + 0.000375 \cdot A^{2} + 0.000272 \cdot B^{2} - 0.035262 \cdot C^{2} \hfill \\ \end{gathered}$$

4 $$\begin{gathered} ln\left( {R2} \right) = 1.00349 + 0.127853 \cdot A - 0.035054 \cdot B + 0.361806 \cdot C + 0.001874 \cdot AB \hfill \\ \quad \quad - 0.038874 \cdot AC + 0.033375 \cdot BC - 0.002709 \cdot A^{2} - 0.001737 \cdot B^{2} \hfill \\ \end{gathered}$$

5 $$\begin{gathered} \frac{1}{{\sqrt[5]{R3}}} = 0.306435 + 0.104288 \cdot A - 0.11052 \cdot B - 0.27082 \cdot C + 0.030481 \cdot BC \hfill \\ \quad \quad - 0.002568 \cdot A^{2} + 0.002484 \cdot B^{2} \hfill \\ \end{gathered}$$

where *A* is the silica fume/binder ratio, *B* is the fly ash/binder ratio, and *C* is the volume content of steel fiber.

### Effects of parameters on mechanical properties and crack sensitivity of sprayed RPC

#### Compressive strength

The variance analysis results of the compressive strength model based on the BBD design are shown in Table 5, and the model equation is given by Eq. (3). The *p*-values of each factor obtained from ANOVA showed that the influence degree of each factor on compressive strength was in the following order: A^2^ > C^2^ > B^2^ > B > AC > C > A. Among them, A^2^, C^2^and B^2^ were significant items, A had an interactive relationship with C.

**Figure 4:**
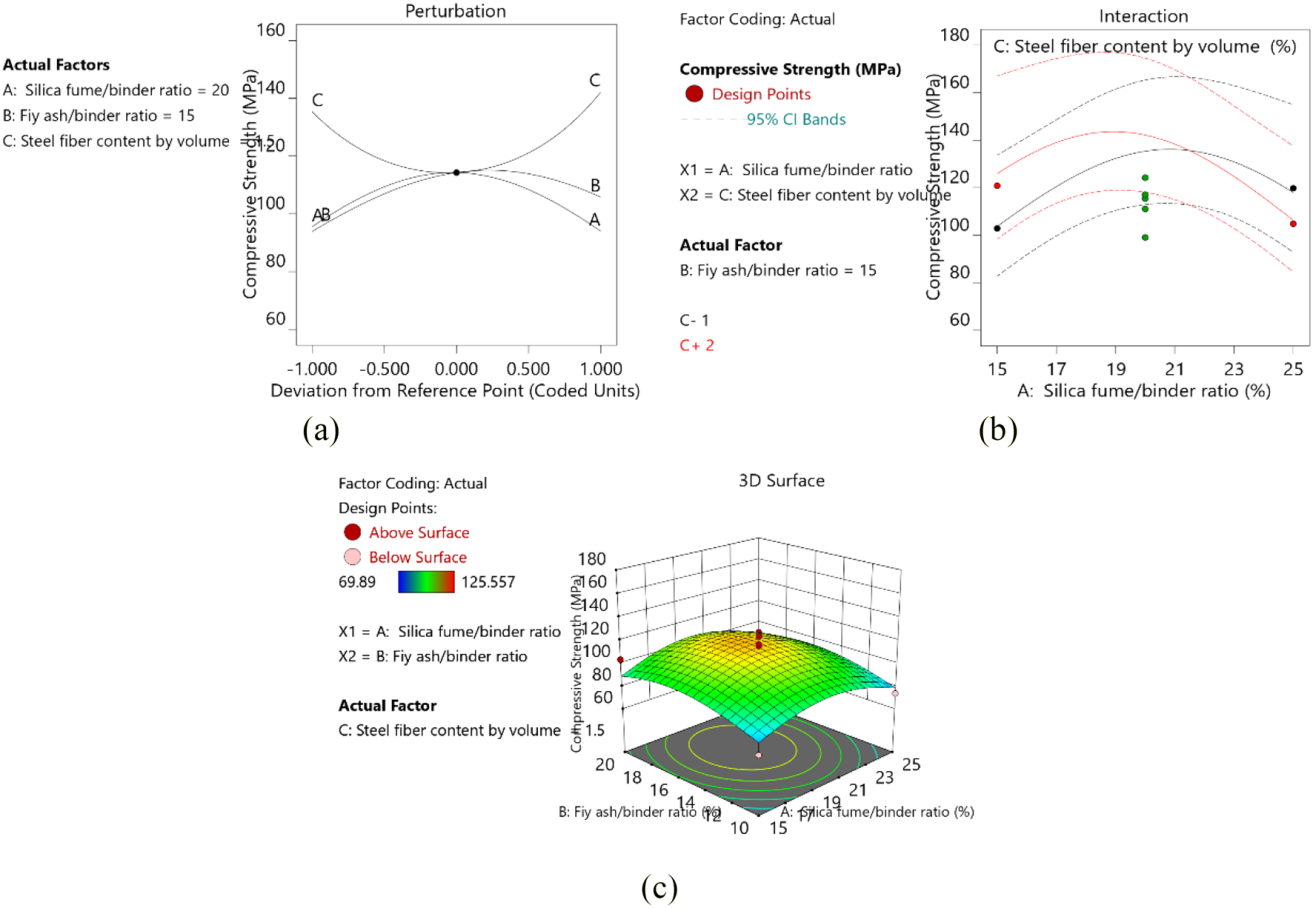
(**a**) Perturbation plot for compressive strength. (**b**) AC interaction, B = 15. (**c**) 3D response diagram of compressive strength.

Figure 4a shows the perturbation plot for the response model of compressive strength. The result showed the variations of the compressive strength at different variables. The compressive strength increased first and then decreased with increasing silica fume/binder and fly ash/binder ratios; it decreased first and then increased with increasing content of steel fiber. Silica fume can densify and homogenize the microstructure of RPC due to its filling effect and the high pozzolanic reaction with CH to form C–S–H gel. Fly ash produced a “ball effect” in the process of stirring, which played a role in filling the gap of particles and increasing the density of slurry to improve the compressive strength of the matrix. However, the loosening effect and the increased viscosity associated with relatively high silica fume content led to the entrapment of air bubbles and eventually lowered the density of the materials^16^.

The strength of steel fibers was higher than that of sprayed RPC matrix and acted as a bridge in the matrix hinders the development of cracks, and improves the compressive strength of sprayed RPC. When the steel fiber content initially increased, the compressive strength of sprayed RPC gradually decreased, which was different from the conclusion of previous RPC studies^11^. This may be because with the increase of steel fiber content, the workability of sprayed RPC decreases, which leads to the decrease of compactness and affects the compressive strength of sprayed RPC. As the steel fiber content continued to increase, the average distance between the fibers decreased. Thus, more fibers bear the load, resulting in the reduction of the stress between the fibers and the matrix, delaying crack formation and propagation, and increasing the strength.

Figure 4b shows the influence of the interaction between A and C on the compressive strength of sprayed RPC. The figure shows that when A < 20, the slope of the red line is smaller than the black line, indicating that the improvement degree of compressive strength is more sensitive to the steel fiber content of 1. The three-dimensional (3D) plot in Fig. 4c within the design boundary showed that the response surface figure had a clear peak, the corresponding contour plot had the highest point, indicating that maximum compressive strength can be achieved within the design boundary. The strength of sprayed RPC is lower than that of ordinary RPC, and the influence trend of steel fiber on these two materials is different, this phenomenon may be caused by the injection process. However, the maximum compressive strength of sprayed RPC exceeds 120 MPa, which meets the requirements of traditional RPC for compressive strength. The compressive strength of sprayed RPC is 71%-140% higher than that of traditional concrete (50–70 MPa)^4,40^.

#### Splitting tensile strength

The splitting tensile strength prediction model designed according to BBD method is shown in Table 5, and the model equation is shown in Eq. (4). According to the p-values of each factor obtained from the ANOVA, the degree of influence of each factor on the splitting strength was in the following order: AC > BC > A > A^2^ > B > C > AB > B^2^. AC and BC were significant mode items, and the others were not significant mode items.

**Figure 5:**
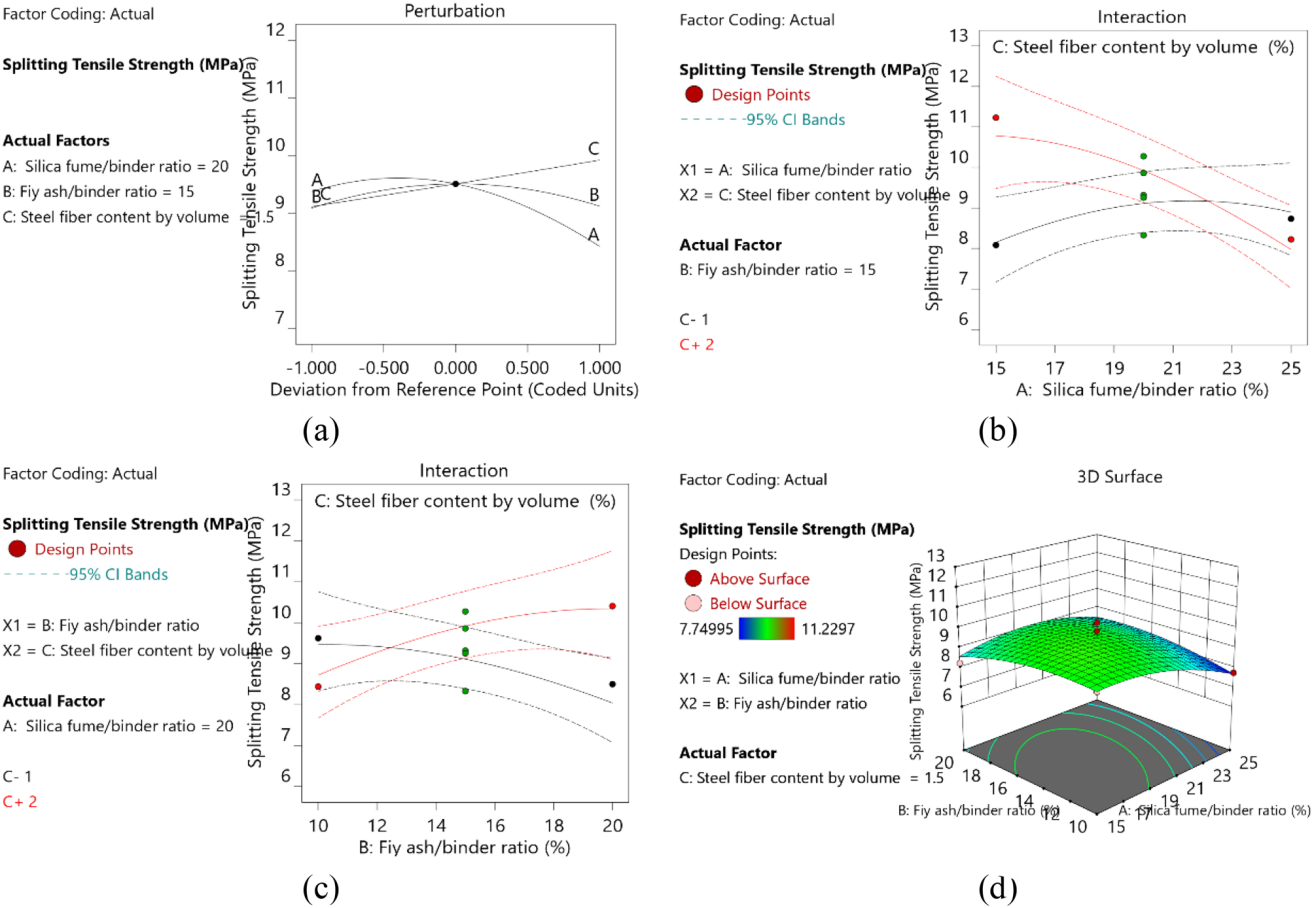
(**a**) Perturbation plot for split tensile strength (**b**) AC interaction, B = 15 (**c**) BC interaction, A = 20 (**d**) 3D response diagram of split tensile strength.

Figure 5a is the disturbance plot of the splitting tensile strength response model. The figure shows that the splitting tensile strength increased linearly with increasing steel fiber content, and it increased first and then decreased with increasing silica fume/binder and fly ash/binder ratios. This finding was due to the fact that the pozzolanic reaction of silica fume increased the content of C-S–H gel, decreased the content of CH, improved the microstructure, and significantly improved the bonding strength between the fibers and the matrix. The bond strength increased gradually as the silica powder increased from 0 to 15% and remained relatively stable when the silica fume content was 20%. When the content of silica fume was further increased to 25%, the bond strength decreased^17^. The bond strength of the fiber-matrix interface was provided by a chemical bond, which anchored mechanical force and friction forces related to fiber ends. As the bond strength decreased, the fibers were more likely to be pulled out from the matrix, and the splitting tensile strength decreased.

The slope of A in Fig. 5a was greater than that of B, which indicated that the splitting tensile strength was more sensitive to silica fume/binder ratio than to fly ash/binder ratio. As important model items, the influence of AC and BC had on the splittiing tensile strength of specimens was shown in Figs. 5b, c. With the change of steel fiber content (C), the influence of silica fume/binder ratio (*A*) and fly ash/binder ratio (*B*) on the splitting tensile strength showed different trends. When C = 1, the splitting tensile strength increased with increasing silica fume/binder ratio and decreased with increasing fly ash/binder ratio. When C = 2, the splitting tensile strength decreased with increasing silica fume/binder ratio, but increased with increasing fly ash/binder ratio. A clear highest point can be seen from the 3D plot in Fig. 5d, indicating that the maximum split tensile strength can be obtained within the range of variable design.

#### Crack sensitivity test

The results of ANOVA of BBD-based crack sensitivity model are shown in Table 5, and the model equation is given by Eq. (5). According to the p-values of each factor obtained from ANOVA, the influence degree of each factor on crack sensitivity was in the following order: C > BC > B > A^2^ > B^2^ > A. C, BC, B, A^2^, and B^2^ were significant terms, A was an insignificant term, and B had an interactive relationship with C.

**Figure 6:**
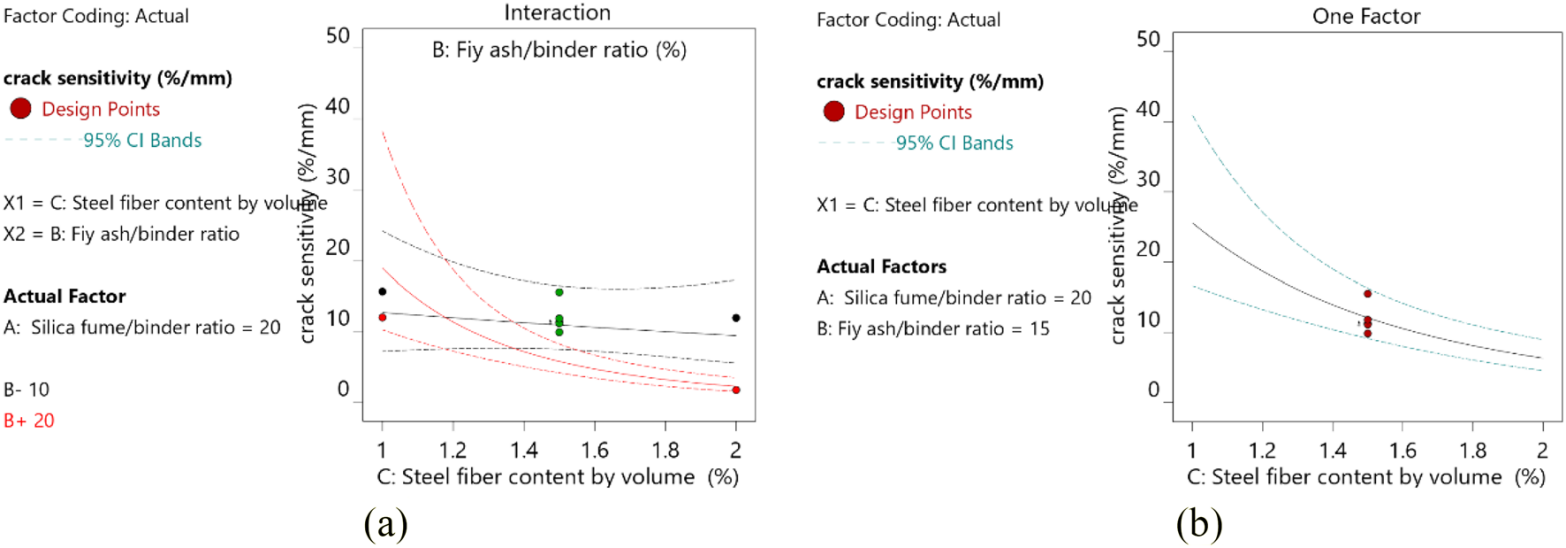
(**a**) Diagram of steel fiber content and compressive strength (**b**) diagram of interaction between B and C, A = 20.

According to p-value, steel fiber content had the most significant effect on crack sensitivity, which was far greater than other factors. Steel fiber was conductive; thus, current was transmitted through contacts between steel fibers or electrical tunnels forming conductive paths. Figure 6a shows that the crack sensitivity decreased with increasing number of steel fibers. This finding was different from those obtained in previous studies^31^, and such difference may be due to the addition of too many steel fibers in sprayed RPC, making it difficult to stir and leading to uneven fiber distribution. When RPC was sprayed into the mold, the gap around the fiber increased, thereby reducing the electrical contact between the fiber and the matrix. As a result, the crack sensitivity decreased with increasing steel fiber content. Figure 6b also shows the interaction between B and C. When C = 10, the content of steel fiber had little effect on crack sensitivity. When C = 20, the crack sensitivity decreased sharply at first and then decreased slowly with increasing steel fiber content.

**Figure 7:**
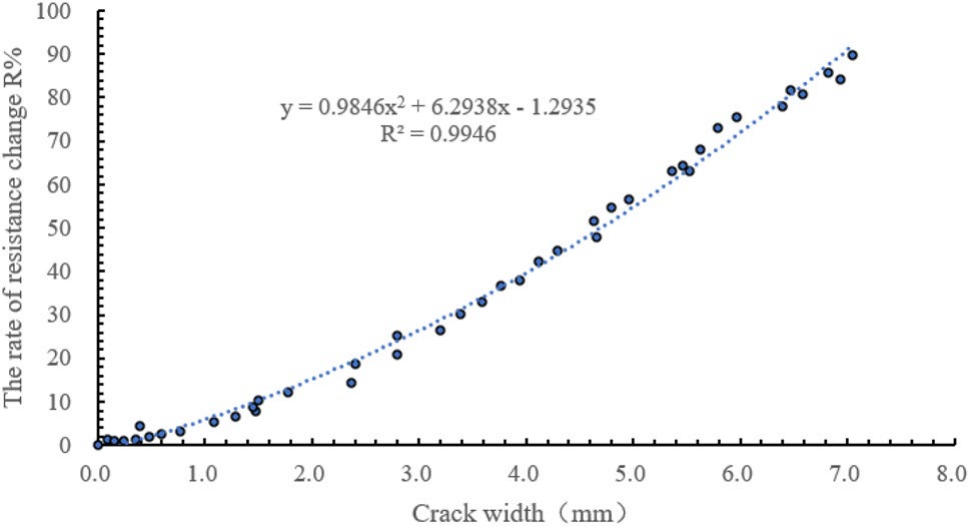
Curve of resistance rate and crack width.

All concrete samples showed similar trends in crack sensitivity, so one group was selected for analysis. Figure 7 shows the relationship between crack width and the rate of resistance change when A = 15, B = 10, and C = 1.5. The figure shows that the resistance change was not obvious before the crack appeared, but when the crack appeared, the resistance change rate began to change. With increasing size of the crack, the rate of resistance change also increased gradually. This was because with increasing crack width, the conductive path formed between the fiber and the matrix was gradually broken, and the tunnel effect was weakened, leading to the increase of resistance.

### Multi-objective optimization of response

Table 6 shows the standard setting of variable multi-objective optimization using response surface method to obtain the best independent variable setting to optimize the best mechanical properties and crack sensitivity of sprayed RPC. The variables were optimized synchronously based on the expectation function, and the optimal value of the independent variables was obtained when the silica fume/binder ratio was 15%, the fly ash/binder ratio was13.203%, and the volume content of steel fiber was 2%. The optimal response value could be obtained under this mixture ratio. Specific predictive values are shown in Table 7. These values were achieved at a desirability value of 75.4%. The target values may be set with different weights and importance depending on the expected setting of the target response. The compressive strength was set in a range, because the optimized compressive strength of 119.107 MPa met the requirements of traditional RPC.

**Table 6:** Optimized standard Settings.

Name	Unit	Goal	Lower Limit	Upper Limit	Importance
A: Silica fume/binder ratio	%	Is in range	15	25	3
B: Fly ash/binder ratio	%	vs in range	10	20	3
C: Steel fiber content by volume	%	Is in range	1	2	3
Compressive strength	MPa	Is in range	69.89	125.557	3
Splitting tensile strength	MPa	Maximize	7.74995	11.2297	5
Crack sensitivity	% mm^-1^	Maximize	1.68129	46.268	5

**Table 7:** Optimal solution of the prediction model.

Response	Compressive strength (MPa)	Splitting tensile strength (MPa)	Crack sensitivity (% mm^-1^)	Desirability
Predictive value	119.107	10.548	12.611	0.754

## Conclusion

In this study, the spray performance of sprayed RPC was studied. Response surface method was used to study the effects of silica fume content, fly ash content and steel fiber content on the compressive strength, splitting tensile strength and crack sensitivity of sprayed RPC. The material mix ratio of sprayed RPC was optimized by multi-objective optimization. The main findings obtained in this work may be found hereafter:

1. According to the test, the optimal content of water reducing agent was 1.1% of cementing material mass. When the fluidity was controlled between 140 and 160 mm, the spray effect of the mixture and the adhesion effect on large plate were better.

2. The models of compressive strength, splitting tensile strength and crack sensitivity were established by Box-Behnken method. ANOVA verified that all models had sufficient accuracy, which could provide theoretical basis for the practical application of sprayed RPC.

3. The model established by ANOVA showed that the steel fiber content had little effect on compressive strength but had a significant effect on other responses. It can also be analyzed from the model that the influence trend of steel fiber on the compressive strength of sprayed RPC is different from that of ordinary RPC, which may be caused by different construction technology. The content of steel fiber was positively correlated with the splitting tensile strength, whereas the crack sensitivity decreased with increasing content of steel fiber.

4. The compressive strength of sprayed RPC can reach 120 MPa, although it is slightly smaller than ordinary RPC, which may be caused by the difference in construction technology, it meets the requirements of RPC for compressive strength, and the compressive strength of shotcrete RPC is increased by 71%-140% compared with traditional shotcrete.

5. Although silica fume and fly ash can significantly improve the mechanical properties of sprayed RPC, excessive silica fume and fly ash have a negative effect on the mechanical properties. And the fly ash/binder ratio will affect the degree of crack sensitivity changing with the steel fiber content to some extent.

6. Based on the multi-objective optimization technology, the best silica fume/binder ratio (A) was 15%, the best fly ash/binder ratio (B) was13.203%, and the best steel fiber volume content (C) was 2%. When the desirability value was 75.4%, the optimal compressive strength, splitting tensile strength and crack sensitivity of sprayed RPC curing for 28d were 119.107 MPa, 10.548 MPa and 12.611% mm^-1^ respectively.

## Some perspectives for future research

In this paper, the basic mechanical performances and ejection properties of sprayed RPC are studied. However, there are still a lot of other performance to be studied in sprayed RPC:

1. Since water has a great influence on the jet performance of sprayed RPC, it is very important to analyze the influence of water-binder ratio on sprayed RPC.
2. As fire is one of the potential risks of most structures, it is very important to study the high temperature resistance of sprayed RPC.
